# Assessing the Emergent Public Health Concern of All-Terrain Vehicle Injuries in Rural and Agricultural Environments: Initial Review of Available National Datasets in the United States

**DOI:** 10.2196/15477

**Published:** 2020-05-29

**Authors:** Bryan Weichelt, Serap Gorucu, Charles Jennissen, Gerene Denning, Stephen Oesch

**Affiliations:** 1 National Farm Medicine Center Marshfield Clinic Research Institute Marshfield Clinic Health System Marshfield, WI United States; 2 Department of Agricultural and Biological Engineering Penn State University University Park, PA United States; 3 Department of Emergency Medicine University of Iowa Carver College of Medicine Iowa City, IA United States; 4 SLO, LLC Bethesda, MD United States

**Keywords:** agriculture, all-terrain vehicle, fatality, injury, off-road vehicle, rural

## Abstract

**Background:**

Injuries related to the operation of off-road vehicles (ORVs), including all-terrain vehicles (ATVs), continue to be a significant public health concern, especially in rural and agricultural environments. In the United States alone, ATVs have played a role in thousands of fatalities and millions of injuries in the recent decades. However, no known centralized federal surveillance system consistently captures these data. Traditional injury data sources include surveys, police reports, trauma registries, emergency department data, newspaper and online media reports, and state and federal agency databases.

**Objective:**

The objectives of this study paper were to (1) identify published articles on ORV-related injuries and deaths that used large databases and determine the types of datasets that were used, (2) examine and describe several national US-based surveillance systems that capture ORV-related injuries and fatalities, and (3) promote and provide support for the establishment of a federally-funded agricultural injury surveillance system.

**Methods:**

In this study, we examined several national United States–based injury datasets, including the web-based AgInjuryNews, the Fatality Analysis Reporting System, databases compiled by the US Consumer Product Safety Commission, and the National Fatality Review Case Reporting System.

**Results:**

Our review found that these data sources cannot provide a complete picture of the incidents or the circumstantial details needed to effectively inform ORV injury prevention efforts. This is particularly true with regard to ORV-related injuries in agricultural production.

**Conclusions:**

We encourage the establishment of a federally funded national agricultural injury surveillance system. However, in lieu of this, use of multiple data sources will be necessary to provide a more complete picture of ORV- and other agriculture-related injuries and fatalities.

## Introduction

### Background

The group of vehicles generically referred to as off-road vehicles (ORVs) are gasoline- or diesel-powered motor vehicles designed to be used on a wide variety of off-road surfaces, including packed or loose dirt, rocks, sand dunes, snow, and marshlands. They typically have large low-pressure tires with knobby treads to grab off-road terrains. Vehicles equipped for use on sand dunes often have tires with paddle-like treads.

**Figure 1:**
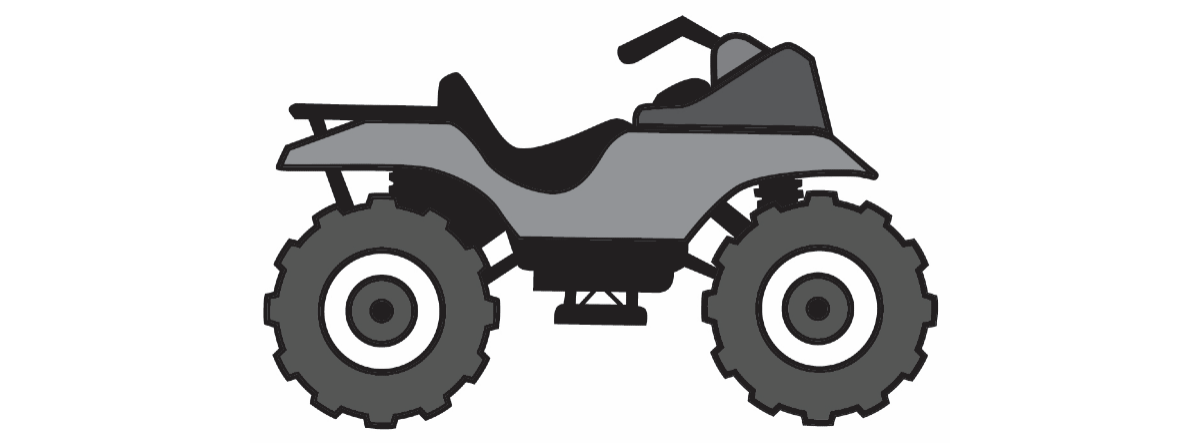
All-terrain vehicle.

A popular ORV, which has been available since the 1970s, is the all-terrain vehicle (ATV; Figure 1). On ATVs, the rider straddles a motorcycle-like seat and uses handlebars to steer, brake, or accelerate. In many other countries, these vehicles are referred to as quads or quad bikes. ATVs have a narrow track width (distance between the middle of the right and left tires), a short wheelbase (distance between the axle or center point of the front and rear wheels), and a high center of gravity. Together, these result in low stability. Given their design, an ATV operator is required to use *active riding*, which involves the operator moving their pelvis and torso laterally and/or longitudinally on the seat, or vertically off the seat, while keeping both hands on the handlebars and both feet on the footrests throughout a maneuver, thus increasing the stability of the ATV and reducing the chance of a rollover [1].

**Figure 2:**
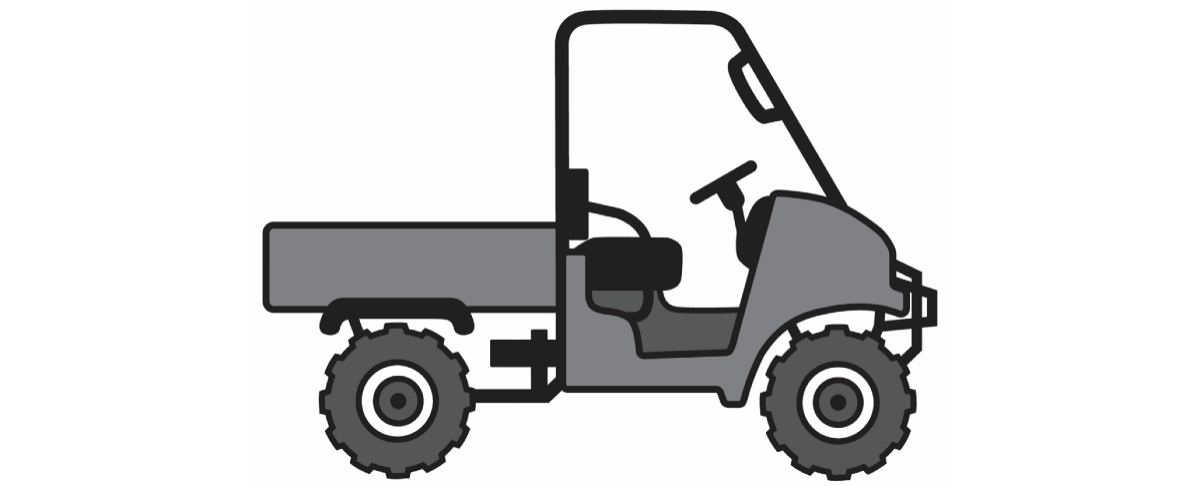
Recreational off-highway vehicle.

A second type of ORV that has gained increasing popularity over the past few decades is generically called a side-by-side (S×S). This includes recreational off-highway vehicles (ROVs; Figure 2) and utility task/terrain vehicles (UTVs). ROVs and UTVs have automobile-like bench or bucket seats, a steering wheel, and foot pedals to activate the brake and accelerator. Some people refer to all S×Ss as UTVs, but, technically, light utility vehicles have maximum speeds of 25 mph, whereas all ROVs are capable of traveling greater than 30 mph [2]. Owing to this, ROVs are required in the United States to have rollover protective structures (ROPS) and restraint devices such as seat belts or a harness system [3]. Although some UTVs have ROPS, many do not.

### Off-Road Vehicle Related Exposure and Injury

Both ATVs and S×Ss are used for a wide range of recreational activities, such as trail and dune riding, hunting, and fishing, and occupational activities, such as forestry, farming, and ranching. In the spring of 2017, an estimated 10.5 million households owned an ATV (with many households owning more than one vehicle), and an additional 2.4 million reported that they intended to purchase an ATV within the next year [4]. This compares with an estimated 5.6 million ATVs in use in 2001 [5]. There is no similar publicly available data on the total number of S×Ss in the United States. However, in its annual report filed with the US Securities and Exchange Commission, Polaris Industries estimated that during 2016, the worldwide sales of ATVs were approximately 400,000 vehicles and of ROVs were about 480,000 [6]. These data also show that the ORV market has been shifting from ATVs to ROVs in recent years.

Loss of control is a common risk resulting in traumatic injuries among ATV operators and passengers. Factors that increase the risk include younger age, being a male driver, inexperience, riding the wrong size ATV, carrying passengers, riding on the road, lack of helmets, and alcohol use [7]. ATVs have been implicated in 15,250 deaths between 1982 and 2017 in the United States [8]. During that period, 22% of the deaths were of children aged under 16 years, with 44% of those younger than 12 years [8]. Most of the deaths and injuries to youth (95%) occur in adult-size vehicles, which they are neither supposed to operate nor ride as passengers [5,9-11]. In fact, more children aged under 16 years in the United States die from ATVs than from bicycle crashes [12], and they have 12 times higher risk of injury than older adults [13]. Furthermore, the economic costs of morbidity and mortality from ATV crashes are high [12].

### Public Roadways

Despite ORVs being designed for off-road use only and manufacturers warning that the vehicles should not be used on public roads, most ATV fatalities occur on roadways [7,14,15]. Between 2004 and 2013, the National Highway Traffic Safety Administration (NHTSA) reported that ATV-related fatalities in their Fatality Analysis Reporting System (FARS) ranged from a low of 307 in 2012 to a high of 381 in 2008. Data from 2017 showed that 87% of ATV-related roadway deaths were of drivers [16]. Helmet use was low, with only 9% wearing helmets. Most of the deaths occurred in single-vehicle crashes (71%) and in rural areas (79%). In addition, 40% of fatally injured ATV operators had a blood alcohol level (BAC) of 0.08 or higher, compared with 28% of motorcycle operators [16].

Surprisingly, despite the trends of injury and death associated with ORVs on public roadways, many municipalities and counties have already enacted or are considering ordinances that would allow open access to ORVs on their roads [17]. Elected officials and law enforcement personnel are often contacted by concerned citizens, health care providers, and injury prevention experts who oppose such legislation. However, more often than not, evidence-based arguments, peer-reviewed literature, testimonials, and media reports have had little to no effect. According to the Consumer Federation of America (CFA), a nonprofit research and education organization, there is a national trend that is gaining greater traction to enact local and state laws to allow ORVs on public roads, with no decline in the foreseeable future [17]. From April 2014 to September 2018, the CFA sent more than 180 letters opposing the use of ORVs on public roads to state and local officials in 27 states, with 40 in the state of Wisconsin alone [18].

### Off-Road Vehicles in Agriculture

Farmers and ranchers were early purchasers and have described ATVs as filling a valuable niche between a truck and a tractor [19]. These versatile machines are leveraged for a variety of agricultural work–related tasks [20]. These include inspecting crops and livestock, tilling, herding animals, spraying pesticides and herbicides, plowing or blowing snow, towing or hauling farm supplies and products, and other general transportation and labor tasks. It is expected that the prevalence of ORV use in farming and ranching, both occupational and recreational, will continue to increase [21].

The incidence of fatal and nonfatal injuries in the Agriculture, Forestry and Fishing industrial sector has become difficult to quantify, particularly after the National Institute for Occupational Safety and Health (NIOSH) discontinued national surveys of nonfatal injuries to self-employed farmers, ranchers, and children on farms. However, the Occupational Safety and Health Administration (OSHA) reported that from 2003 to 2013, there were 2090 injuries and 321 deaths because of occupational use of ATVs, with 60% of ATV-related fatalities occurring in agriculture [22]. The use of ATV-mounted weed sprayer tanks is especially problematic as mounting the tank on the vehicle both raises and shifts the center of gravity, making an already unstable vehicle even more likely to overturn [23].

### Objective

The objectives of this study were to (1) identify published articles on ORV-related injuries and deaths that used large databases and determine the types of datasets that were used, (2) examine and describe several national United States–based surveillance systems that capture ORV-related injuries and fatalities, and (3) promote and provide support for the establishment of a federally funded agricultural injury surveillance system.

## Methods

### Review of Injury Data Sources

An electronic literature search of all articles published between 2014 and 2018 was conducted using PubMed to identify ORV-related articles. The terms used in various combinations in Medical Subject Headings and keyword searches included “off-road vehicles,” “fatalities,” “accidents,” “wounds,” “injuries,” “ATV,” “UTV,” and “mortality.” Our search yielded 70 results, and the abstracts of these articles were reviewed. Published reports were included in this study if they had used a large database of stored information from which they identified ORV-related crashes, injuries, or deaths. A total of 17 articles met the inclusion criteria.

### National Datasets

In this report, 4 national United States–based datasets that include ORV-related crashes and injuries were selected for review: AgInjuryNews, Consumer Product Safety Commission (CPSC), FARS, and the National Fatality Review Case Reporting System (CRS). Although this paper focuses on 4 US databases, there are several other national datasets that include ORV-related injuries and/or deaths. For example, the Bureau of Labor Statistics (BLS) collects the US occupational injury and fatality data. Under the auspices of the BLS, the Census of Fatal Occupational Injuries captures occupational fatalities, including those of volunteers and undocumented workers [24], and the Survey of Occupational Injury and Illnesses database collects nonfatal occupational injuries, including those solicited from agricultural employers having more than 10 employees [24]. The OSHA also investigates work-related fatalities, but excludes self-employed individuals, members of the immediate family of farm employers, and employees of state and local governments. In addition, the OSHA investigation’s inclusion/exclusion and general oversight criteria vary from state to state [25]. Neither BLS nor OSHA captured bystander or passenger injuries, unless the bystander or passenger was also working at the time of the incident. In summary, BLS and OSHA data provide a very limited view of agricultural injuries, and for these reasons, they were not included or further explained in this review.

### Ethics Approval and Informed Consent

No human subjects were involved in this project.

## Results

### Systematic Review

Table 1 summarizes the 17 ORV-related articles published between 2014 and 2018 that used large datasets. Most researchers have used trauma center and/or emergency department (ED) data as well as data from the FARS; the CPSC, including their National Electronic Injury Surveillance System (NEISS); and state departments of transportation (DOTs). These studies primarily focused on demographics, severity of injury, body part injured, and risk factors. However, sources that included a variety of vehicle- and crash-related variables rarely indicated whether the vehicle was used for recreational or occupational purposes [26].

**Table 1:** Summary of off-road vehicle–related studies (2014-2018) and the data sources used.

Reference (year)	Data source	Study period	Study populations
Richardson et al (2018) [27]	FARS^a^, CPSC^b^, and vehicle sales database	2000-2015	All age groups
Nabaweesi et al (2018) [28]	National Emergency Department Sample	2006-2011	Pediatric (0-17 years)
Karkenny et al (2018) [29]	NEISS^c^	1991-2014	2-18 years
Testerman et al (2018) [30]	Level I trauma center	2005-2015	All age groups
Nolan et al (2018) [31]	Level I trauma center	1999-2005	All age groups
Flaherty et al (2017) [32]	Massachusetts emergency departments	2002-2013	Pediatric (0-17 years)
Benham et al (2017) [33]	Level I trauma center	2008-2012	Adult and pediatric
Lombardo et al (2017) [34]	NEISS	2007-2012	Pediatric (0-17 years)
Garay et al (2017) [35]	Pennsylvania State Trauma Database	2004-2014	Pediatric (0-17 years)
Qin et al (2017) [36]	Iowa Department of Transportation, Department of Natural Resources, and State Trauma Registry	2002-2013	All age groups
Gorucu et al (2017) [37]	Pennsylvania Department of Transportation roadway crash data	2010-2013	All age groups
Linnaus et al (2016) [38]	Level 1 pediatric trauma center	2007-2015	Pediatric (0-17 years)
Bethea et al (2016) [39]	Level 1 trauma center	2005-2013	All age groups
Lagerstorm et al (2016) [40]	CPSC	2011-2013	All age groups
Sciarretta et al (2016) [41]	Level II trauma center	Not available	Pediatric (0-17 years)
Williams et al (2014) [42]	FARS	2007-2011	All age groups
Denning et al (2014) [9]	CPSC	1985-2009	Pediatric (0-17 years)

^a^FARS: Fatality Analysis Reporting System.

^b^CPSC: Consumer Product Safety Commission.

^c^NEISS: National Electronic Injury Surveillance System.

### National Datasets for Off-Road Vehicle–Related Injuries and Deaths

Similar to many other subsectors of injury prevention and injury epidemiology, there is a lack of a comprehensive national injury surveillance system for ORV-related injuries, including those from agricultural use of the vehicle. In the following sections, descriptions of the 4 selected national datasets are provided. Table 2 summarizes the characteristics of these national data sources for ORV-related injuries and deaths.

**Table 2:** Characteristics of national data sources for all-terrain vehicle–related injuries.

Properties	AgInjuryNews	CPSC^a^	Fatality Analysis Reporting System	The National Fatality Review CRS^b^
Responsible organization	National Farm Medicine Center and Marshfield Clinic Research Institute	Independent agency of US government	National Highway Traffic Safety Administration	National Center for Fatality Review and Prevention
Purpose	To provide an interactive display of publicly available injury reports data involving AgFF^c^-related injuries and fatalities	To protect the public against unreasonable risks of injury or death from consumer products through education, safety standards activities, regulation, and enforcement	To provide an overall measure of highway safety, to help suggest solutions, and to help provide an objective basis to evaluate the effectiveness of motor vehicle safety standards and highway safety programs	To promote, support, and enhance fatality review methodology and activities for fetal and infant mortality review and CDR^d^
Inclusion and exclusion criteria	Included: injuries and fatalities related to AgFF; excluded: recreational and non-AgFF cases	Included: consumer product–related injuries evaluated at NEISS^e^ emergency departments and consumer product–related fatalities; excluded: CPSC notes that some states may not report all all-terrain vehicle deaths within their state	Included: fatal traffic crashes involving a motor vehicle on public roadways; excluded: motor vehicle deaths occurring >30 days after the incident	Included: all child deaths reviewed by local review teams in states that utilize the CDR CRS
Data collection period	2015 to present	1982 to present	1975 to present	2005 to present
Primary data sources	News media, social media, obituaries, police reports, and Fatality Assessment and Control Evaluation reports	NEISS, death certificates, in-depth CPSC investigations, news media, and coroner/medical examiner reports	Police crash reports, death certificates, state vehicle registration files, coroner/medical examiner reports, state driver licensing files, hospital medical reports, state highway department data, emergency medical service reports, vital statistics, and other state records	Agencies represented on CDR teams share case-specific information at multidisciplinary meetings. Represented agencies include, but are not limited to, medical examiner or coroner, law enforcement, child protective services, medical providers, and school districts
Data collection methods	News media monitoring service, Google Alerts, and submissions from colleagues and users	Death certificates, news media monitoring, and CPSC crash investigations	State submission of police crash reports and other data	Cases are identified through medical examiners, coroners, and vital records
Crash location–related variables	Location and type of road	Location and type of road	Location, type of road, crash characteristics, environmental conditions, and first harmful event	Location and driving conditions
Vehicle-related variables	Vehicle type	Engine size; vehicle type, make, and model; and the presence of passengers	Vehicle type, make, and model; most harmful event; extent of damage; and vehicle- and driver-level related factors	Child’s vehicle, other primary vehicle, and number of occupants
Victim-related variables	Demographics, operator/passenger, injury severity (fatal/nonfatal), agricultural work relatedness, safety equipment (eg, helmet, seatbelt, and gear), injury event, and injury sources	Demographics, vehicle safety training, operators’ height/weight, and alcohol/drug usage	Demographics, seating position, alcohol/drug usage and test results, fatal injury at work, and safety equipment (eg, helmet, seatbelt, and gear)	Demographics, seating position, causes of incident (eg, speeding and distraction), vehicle safety training, safety equipment (eg, helmet, seatbelt, and gear), and alcohol/drug usage

^a^CPSC: Consumer Product Safety Commission.

^b^CRS: Case Reporting System.

^c^AgFF: agriculture, fishing, and forestry.

^d^CDR: child death review.

^e^NEISS: National Electronic Injury Surveillance System.

#### AgInjuryNews

AgInjuryNews was developed by the National Farm Medicine Center and launched in 2015 [24]. The team responsible for this endeavor compiles AgFF-related injuries and fatalities from publicly available sources such as news media outlets, obituaries, social media, and police reports [24]. This is accomplished through several search platforms, including a media monitoring service, Google Alerts, social media (eg, sheriff departments’ Facebook pages and GoFundMe), and submissions from colleagues [24]. Data are collected, coded, uploaded to the center’s interactive searchable website, and made available for public use. The goal of this repository is to provide a comprehensive list of all deaths and injuries occurring on farms and ranches, including cases involving children as bystanders and/or farm visitors [20]. Data collection methods of the AgInjuryNews initiative are further described in a different paper [43]. Data for this study were available to the authors of this paper through prearranged administrative privileges.

ORV-related injuries occurring on a farm or ranch are included in the AgInjuryNews database. To distinguish occupational ORV-related fatalities, AgInjuryNews researchers use farm and agricultural injury classification (FAIC) codes. FAIC codes provide a systematic scheme for separating farm/agricultural production work cases [44]. It is often difficult to differentiate between occupational and nonoccupational ORV-related cases as there might not be enough detailed information from news reports to use the FAIC. AgInjuryNews researchers often follow-up and try to gather more information to distinguish occupational from nonoccupational cases.

With regard to ORV-related cases, AgInjuryNews uses the Occupational Injury and Illness Classification System (OIICS) for coding the vehicle involved in the injury. There is a specific OIICS code for ATVs (code: 8611), but not for other types of ORVs. AgInjuryNews coders use the OIICS code 8619 (off-road passenger vehicles—powered, not elsewhere classified) for ORVs other than ATVs [45]. Other variables available in the database include demographics of injured victims, crash location (eg, roadway, farm, field, or orchard), whether the incident was work-related or recreational, injury source (eg, vehicle type), event/activity at the time of the incident (transportation, fall, or contact), and others. Detailed information on the available variables can be found in AgInjuryNews [46].

In the past, a collection of news reports could successfully capture nearly all fatal incidents and identify agricultural injury and fatality cases at the local, regional, and national levels [47,48]. The AgInjuryNews initiative has become a systematic, up-to-date, web-based collection of agriculture-related injuries and fatalities that fill a surveillance gap and provide national-level data to guide research, injury prevention efforts, and organizational policy for agribusiness [24]. Media reports collected by AgInjuryNews over time have shown how the ATV-related injury category has quickly risen to the top as a source of injury among youth in agriculture, with ATVs being the second leading cause of nonfatal injuries and the leading cause of fatal injuries among those younger than 18 years [24].

The AgInjuryNews dataset, established in 2015, is limited by the information available in the original sources, which are primarily web-based news media reports [43]. These types of reports likely capture more serious traumatic injuries and fatalities. However, media reports are inherently inconsistent in the type of information they provide. For example, not every journalist asks the same questions, or they may simply redistribute statements from the responding sheriff’s department or fire chief. When the injury is nonfatal, data variables such as age and gender are not always reported. Moreover, journalists often mislabel the various types of ORVs involved, for example, calling an S×S an ATV. Sometimes this error can be identified by other information included in the article, such as the rider not using their seat belt (only available on S×Ss), but not always. To further complicate things, DOT data also vary across states, based on how ATVs and S×Ss are coded. In addition, this dataset may inadvertently include cases that are not agricultural because of the difficulty in identifying vehicle use at the time of the crash.

#### Consumer Product Safety Commission

As ORVs are designed for off-road use only, manufacturers are not regulated by the Federal Motor Vehicle Safety Standards issued by NHTSA for roadway vehicles. Instead, they are regulated by the CPSC, an independent 5-member commission. The CPSC releases an annual report on the deaths and injuries related to ATV use in the United States. There are no comparable annual reports on the deaths and injuries related to S×Ss. On the basis of the cases collected by the CPSC, estimated deaths from the use of ATVs peaked at 923 in 2005 and declined to 651 in 2013. However, the number of fatalities appears to be increasing again, as there were an estimated 708 deaths in 2015 [8].

Data collected by the CPSC on ATV-related fatalities are available to researchers upon request for secondary analyses. This is accomplished by completing and submitting a Freedom of Information Act request form through the CPSC website [49]. The CPSC also prepares estimates of hospital ED-treated injuries related to consumer products through its NEISS, which is a probability sample of EDs in the United States. There were an estimated 93,800 ATV-related injuries treated in EDs in 2017. Of those injuries, the CPSC estimates that 24,800 (26%) were to children under 16 years [8].

Public access to the NEISS is available through the CPSC website, and individuals may view and download the national injury estimates for a multitude of consumer products, including ATVs [50]. NEISS uses 4 different codes for ATVs based on the number of wheels (3, 4, more than 4, and unspecified number of wheels). Through the NEISS Query Builder, customized searches may be performed, and deidentified case data may be downloaded for further analysis. Variables available include demographics (age, sex, and race), product involved, date of injury, general location where the injury occurred, body part injured, diagnosis, and patient disposition. There is also a brief narrative that provides a description of the incident.

The CPSC fatality data are limited with regard to information about crash events and driver actions [42]. Some variables such as information on the make and model of the ATV involved in the crash are restricted and not made available to the public [47]. A major limitation of the CPSC data is that there is a substantial time lag in reporting data on ATV fatalities. For example, the most recent annual report was released in February 2019, but the last year for which ATV fatality data in this report were considered complete was 2014. Data collection was still ongoing at the time of this study for 2015 to 2017 [8].

Although the CPSC began its collection of ATV fatality data in 1982, the agency switched from using death certificate mortality codes under the ninth revision of the International Classification of Diseases to the tenth revision in 1999. The CPSC says that comparisons of pre-1999 data with the later data “should be undertaken with caution” [51]. In addition, CPSC death counts by state reflect the state in which the death occurred rather than the state in which the crash occurred. Using emergency medical services’ air and ground transportation for the most critically injured ATV riders to level 1 trauma centers in other states may inflate the number of deaths reported for a state in which the rider was finally treated. Unfortunately, the CPSC does not actively collect data related to S×Ss and does not include them in their annual ATV death and injury reports.

The NEISS is an easily accessible database that provides probability sampling of national ED data, but it is fairly limited in the information it collects. The mechanism of the crash and injury is not coded, and information regarding key risk factors for ATV crashes and injuries such as helmet use, presence of passengers, vehicle engine size, alcohol and other drug use, and vehicle speed are also not specifically recorded. The short narrative often provides some of this information, but it is not reliably documented. Although the NEISS does have a code for *utility vehicle*, there are no further categorizations in the system for this type of vehicle, and vehicles other than UTVs and ROVs may be coded under this designation.

#### Fatality Analysis Reporting System

NHTSA maintains the FARS, which is a census of and the sole source of all police-reported motor vehicle–related fatalities on public roads in the United States [52]. This data collection system, which was established in 1975, includes both motorists and nonmotorists who die within 30 days of being involved in a motor vehicle traffic crash [53]. Through a cooperative agreement with agencies in each state, NHTSA collects fatality crash data that are converted to the SAS data format. The sources of the FARS data include, among other things, police crash reports, death certificates, and coroner/medical examiner reports [53]. FARS cases are only considered to be work-related if the *injury at work* response item on the death certificate is checked [54].

The FARS query system allows public access to the database. Data may be processed utilizing the site’s interactive user interface, and customized searches may be performed. A create-a-map feature allows individuals to build county-by-county and state-by-state maps displaying personally selected results from the FARS data. Published files may also be downloaded from the FARS website (ftp://ftp.nhtsa.dot.gov/FARS) as compressed delimited text files or SAS data files. Requests for specific data may be made to the NHTSA National Center for Statistics and Analysis at no charge, and it usually takes about 2 weeks depending on the complexity of the data requested.

The FARS data contain more than 100 separately coded elements [53]. In addition to demographic information, the dataset includes a number of variables noted to be risk factors for ATV crash and injury, including helmet use, seating position, presence of passengers, speed, and BAC. The roadway type and type of surface, specific location of the crash on the road, and rural/urban location data are also available. FARS provides a number of variables that distinguish what happened in the crash, including the number and type of vehicles involved; the first harmful event that occurred; the crash configuration and maneuvering of each vehicle involved; and driver-related contributing factors for every vehicle, based on police judgment.

In 2013, the Insurance Institute for Highway Safety, a nonprofit research and communications organization funded by motor vehicle insurers, released the first study to use FARS data to identify the characteristics of on-road fatal ATV crashes [42]. A primary reason for conducting the study was that CPSC data showed that most ATV deaths occurred on public roadways rather than off road [14,15].

The FARS dataset is limited to police-reported fatalities on public roadways and does not include those occurring off road. Moreover, FARS uses body type code 90 for ATVs with 3 or 4 wheels, but S×Ss, including ROVs and UTVs, have also been coded under this body type as well as in the *other vehicle* category. Although one could try to use vehicle identification numbers (VINs) to delineate these ORV types, only about one-half of VINs could be decoded in an ATV study utilizing FARS data [42]. Beginning with its report on 2017 fatalities, NHTSA added a new category, *recreational off-highway vehicles*, to cover ROVs [55,56]. Therefore, this database may be more useful to conduct studies related to ROVs on public roadways in the future. In addition, FARS inclusion requires the person’s death to be within 30 days of the crash, and fatalities occurring beyond this period would be missed.

#### The National Fatality Review Case Reporting System

The National Center for Fatality Review and Prevention (NCFRP) is funded by the Maternal and Child Health Bureau under the Health Resources and Services Administration and is the national resource and data center for fetal and infant mortality review and child death review (CDR) [57]. The NCFRP manages and promotes the use of the National Fatality Review CRS, which is a standardized case report tool made available to all states. Currently, 43 states utilize CRS with over 2100 data users [57].

The National Fatality Review CRS contains more than 2600 variables that describe in detail the risk factors and circumstances surrounding a child’s death. Although each state varies in its data collection process, information for the case report is generally gathered through multidisciplinary team meetings. The case report is deidentified at the national level. Many states will disseminate their CDR findings into reports to educate policy makers and the general public about the key risk factors and opportunities for injury prevention. Researchers may apply to utilize the national dataset for injury prevention studies [58]. NCFRP’s policies and guidelines should be followed by researchers to apply and use their data.

The dataset only includes deaths reviewed by CDR teams, not all child deaths; therefore, it cannot be used to calculate incidence. In addition, case reports are completed by numerous data users, which can lead to variability in data completeness.

## Discussion

### Principal Findings

A plethora of published research shows that ATV-related deaths and injuries are a significant and ongoing public health concern, including in rural areas and among youth. Although few studies of S×S-related crashes are available, data suggest that injuries and deaths associated with them are an emerging public health issue. Ongoing research is critically needed, including research on agricultural ORV injury prevention.

One of the greatest challenges to ORV-related research is the lack of a single comprehensive data source for fatalities and injuries. This review of some national databases providing information on ORV-related deaths and injuries demonstrates that each database has significant limitations, especially regarding the ability to distinguish recreational from occupational crashes.

Although AgInjuryNews provides agricultural work–related information, it is unable to provide a comprehensive picture of all ORV-related crashes on farms and ranches, as not all rural injuries will be covered by media. Despite this, state and regional efforts to collect media related to agricultural injuries and deaths have grown in number. There are no federally supported comprehensive national databases on agricultural work–related deaths and injuries; such efforts provide some insight into work-related injuries and emerging issues to those involved in agricultural injury prevention. As AgInjuryNews collects data nationally and makes it available to the public, it may supersede more localized efforts and become increasingly more important as a supplemental surveillance system to study agricultural work–related injuries and deaths, including those because of ORVs.

The CPSC ATV fatality database provides information on most, but not all, ATV-related fatalities in the nation. Although it codes whether the activity at the time of the crash was work-related, there is little additional information, and it is not possible to determine if it occurred while agricultural work was being performed.

The NEISS database, also maintained by the CPSC, does not specify whether the injury was work-related, limiting its utility for the study of agricultural work–related ATV injuries. As a part of its proposed rulemaking in 2009, designed to create an improved safety standard for ROVs, CPSC released studies on S×S-related fatalities and injuries [3]. However, CPSC has not updated that information to cover more recent years, and the CPSC does not publish an annual report on ROV-related deaths and injuries, similar to what they do for ATVs.

The FARS database provides a great deal of information regarding ORV-related roadway fatalities; however, ORVs are designed to be used off road, and there are a substantial number of fatalities that are not included. In addition, FARS identifies work-related fatalities using only the *injury at work* item on the death certificate, and it does not indicate what type of work activities were being performed at the time of the incident. Thus, identifying agricultural work–related fatalities is not possible using FARS data. As noted previously, with the new code of ROV added in 2017, FARS data could be used to study ROV roadway crashes in the future.

National Fatality Review CRS data comprise many details regarding the mechanisms and activities that were being performed at the time of the fatal crash. This includes the vehicles involved in the incident, including ORVs, and information regarding whether work was being performed. Unfortunately, not all child deaths fall under the auspices of a state or county CDR team. Thus, the CRS data may be rich in detail but may not provide accurate total counts of ORV-related deaths. Moreover, not all states participate in the system. These identified gaps not only hinder the lines of inquiry but also highlight important future work for the discipline.

Although there are challenges in law enforcement, the passage of laws (such as those requiring helmet use while riding ORVs and seat belt use while riding in ROVs) is another important method to reduce the frequency and severity of injuries when ORVs crash [59-64]. Moreover, crash and injury prevention measures found to be effective should be replicated across the country. Given the prevalence of ORV-related injuries and deaths, solutions beyond traditional approaches to improve the health and safety of rural ORV operators need to be found and disseminated.

There is also an upward trend of municipalities permitting the use of ORVs on paved and unpaved public roads to appease constituents, power sport dealerships, and ORV clubs. Active efforts by individuals and community groups are greatly needed to affect decision making and ordinances at the local level [65]. Safety and health professionals and associated organizations should advocate for policy change at the state and national levels, and policy makers need to be made more aware of the issue, encouraged to pass evidence-based safety laws, and discouraged from passing laws that decrease safety, such as opening public roadways to recreational use of ORVs [14,15,42]. Such efforts need to be considered a priority by injury prevention stakeholders, including legislators, to address this growing public health concern.

### Conclusions

Deficiencies in national agricultural injury surveillance efforts continue to plague subsectors of injury prevention research and practice, including efforts to reduce ORV-related injuries. Our review provides illustrations of how the currently available datasets used to perform agricultural ORV-related injury surveillance, and in fact, all agricultural injury surveillance, are inadequate. Significant limitations exist for both individual data sources and even, where possible, merged data from multiple sources. These limitations provide a strong rationale for a robust national surveillance system for agricultural deaths and injuries, which could facilitate the development and evaluation of injury prevention approaches, including evidence-based safety engineering and legislation. A discussion of what such a surveillance system should look like and how it should operate is highly complex and, thus, beyond the scope of this review. However, robust injury surveillance is an essential element in successful efforts to save lives, protect health, and reduce the high costs of preventable injuries.

## Abbreviations

AgFF: agriculture, fishing, and forestry
ATV: all-terrain vehicle
BAC: blood alcohol level
BLS: Bureau of Labor Statistics
CDR: child death review
CFA: Consumer Federation of America
CPSC: Consumer Product Safety Commission
CRS: Case Reporting System
DOT: department of transportation
ED: emergency department
FAIC: farm and agricultural injury classification
FARS: Fatality Analysis Reporting System
NCFRP: National Center for Fatality Review and Prevention
NEISS: National Electronic Injury Surveillance System
NHTSA: National Highway Traffic Safety Administration
NIOSH: National Institute for Occupational Safety and Health
OIICS: Occupational Injury and Illness Classification System
ORV: off-road vehicle
OSHA: Occupational Safety and Health Administration
ROPS: rollover protective structures
ROV: recreational off-highway vehicle
S×S: side-by-side
UTV: utility task/terrain vehicle
VIN: vehicle identification number
